# Loss of chromosome cytoband 13q14.2 orchestrates breast cancer pathogenesis and drug response

**DOI:** 10.1186/s13058-024-01924-4

**Published:** 2024-11-27

**Authors:** Parastoo Shahrouzi, Youness Azimzade, Wioletta Brankiewicz-Kopcinska, Sugandha Bhatia, David Kunke, Derek Richard, Xavier Tekpli, Vessela N. Kristensen, Pascal H. G. Duijf

**Affiliations:** 1 Department of Medical Genetics, Institute of Clinical Medicine, Faculty of Medicine, University of Oslo and Oslo University Hospital, Oslo, Norway; 2 Oslo Center for Biostatistics and Epidemiology, University of Oslo, Oslo, Norway; 3 School of Biomedical Sciences, Centre for Genomics and Personalised Health at the Translational Research Institute, Queensland University of Technology (QUT), Woolloongabba, QLD 4102 Australia; 4 Faculty of Health, School of Biomedical Sciences, Queensland University of Technology (QUT), Woolloongabba,, QLD 4102 Australia; 5 Centre for Cancer Biology, Clinical and Health Sciences, University of South Australia, Adelaide, SA Australia

**Keywords:** Copy number alterations, Chromosome 13q14.2, Breast cancer, Biomarker, Drug sensitivity, Tumor microenvironment

## Abstract

Breast cancer (BCa) is a major global health challenge. The BCa genome often carries extensive somatic copy number alterations (CNAs), including gains/amplifications and losses/deletions. These CNAs significantly affect tumor development, drug response and patient survival. However, how individual CNAs contribute is mostly elusive. We identified loss of chromosome 13q14.2 as a key CNA in BCa, occurring in up to 63% of patients, depending on the subtype, and correlating with poor survival. Through multi-omics and in vitro analyses, we uncover a paradoxical role of 13q14.2 loss, promoting both cell cycle and pro-apoptotic pathways in cancer cells, while also associating with increased NK cell and macrophage populations in the tumor microenvironment. Notably, 13q14.2 loss increases BCa susceptibility to BCL2 inhibitors, both in vitro and in patient-derived xenografts. Thus, 13q14.2 loss could serve as a biomarker for BCa prognosis and treatment, potentially improving outcomes for BCa patients.

**Graphical abstract:**

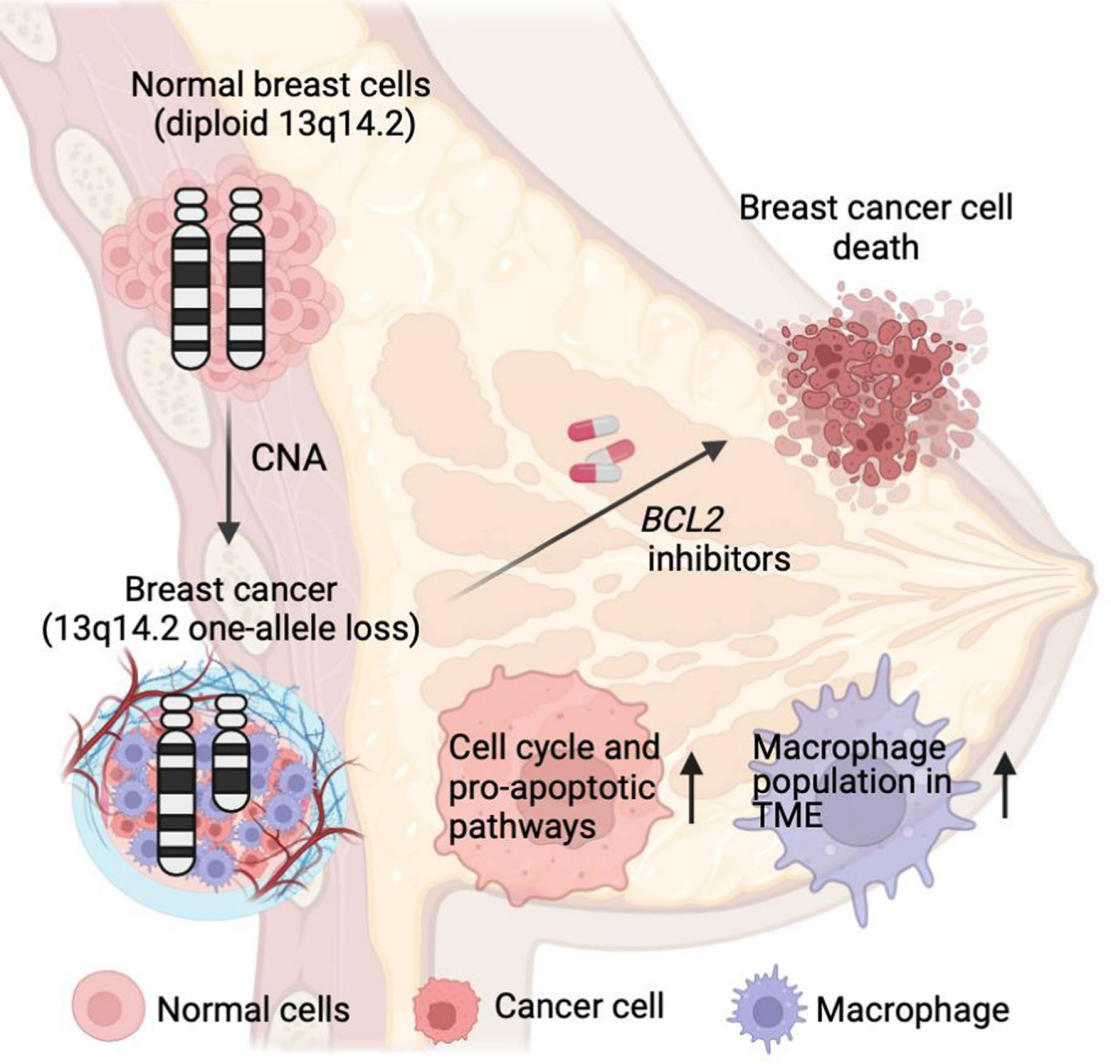

**Supplementary Information:**

The online version contains supplementary material available at 10.1186/s13058-024-01924-4.

## Introduction

Breast cancer (BCa) remains a prevalent global concern, affecting approximately one in eight women and one in 726 men throughout their lifetime [1]. The current classification of BCas in subtypes mostly relies on the expression and genomic presence or absence of individual or a group of genes. While this has advanced therapy selection [2], significant heterogeneity observed within these defined subtypes often results in inadequate treatment response and the development of drug resistance. In addition, chemotherapy is associated with severe side effects in BCa [3]. Together, this underscores the critical need for identifying new biomarkers to enable innovative patient stratification and to improve treatment approaches.

Tumor heterogeneity in BCa is partly influenced by the acquisition of genomic abnormalities, such as CNAs [3, 4]. Errors in DNA damage repair processes or mitosis contribute to CIN, which in turn leads to the formation of CNAs. CNAs involve the gain/amplification and loss/deletion of entire chromosomes/chromosome arms or smaller genomic regions, resulting in aneuploidy and focal CNAs, respectively [4, 5]. Many studies have focused on gene-level CNAs, such as those affecting *MYC*, located on chromosome 8q24, widely recognized as the *MYC* amplicon [6–9]. Our recent research has uncovered a significant functional association between *TRIB1* and *cMYC*, arising through a co-amplification mechanism due to their proximity on the 8q24 locus [10]. The co-amplification of other genes in this region alongside c*MYC* could have collective effects on tumor biology. This underscores the importance of exploring the broad phenotypic impacts of CNAs beyond individually well-characterized genes.

Chromosomal cytoband 13q14.2 encompasses 67 genes, including 28 protein-coding and 39 non-coding genes [11], making it a region of considerable genomic importance. Notably, loss of this region represents the most common CNA observed in chronic lymphocytic leukemia (CLL) [12–14]. In BCa, the focus has been predominantly on mutations or loss of single genes within the 13q14.2 region, such as *Retinoblastoma 1 (RB1)* [15, 16]. However, the potential role of 13q14.2 loss in breast tumors and its impact on the TME beyond *RB1* loss remains largely unexplored.

While CNAs under positive selection are often drivers of oncogenesis, their presence may not always confer an advantage in the context of anti-cancer therapies. We recently reviewed the dual role of CNAs in drug response, elucidating their potential as drivers of both drug sensitivity and drug resistance [4]. The concept of drug susceptibility conferred by specific CNAs is relatively unexplored but holds significant promise for leveraging existing drugs effectively.

The anti-apoptotic BCL2 protein was first identified in 1984 [17]. Thirty-two years later, the Unites States Food and Drug Administration (FDA) approved the BCL2 inhibitor venetoclax for treating aggressive CLL [18]. Overexpression of *BCL2* in 73% of BCas, with 86% in *estrogen receptor-positive (ER*+*)* tumors [19], nominates BCa as the first non-hematologic cancer type to be targeted with BCL2 inhibitors. Currently, the PALVEN clinical trial evaluates the efficacy of venetoclax in combination with the cell cycle inhibitor palbociclib and the aromatase inhibitor letrozole in *ER*+, *human epidermal growth factor receptor 2-negative (HER2-)*, and *BCL2*+ BCa patients [20]. While venetoclax has shown promising induction of cell death in hematological malignancies [17], improving treatment response to this drug in BCa may enhance the success of clinical trials in two ways: 1. by utilizing combination therapy to overcome resistance to single therapies and 2. by selecting patients whose cancers are most likely to respond to the drug.

In this study, we hypothesized that loss of 13q14.2 is a significant pathological event in BCa that also influences drug response. To test this, we first investigated the prevalence of 13q14.2 loss in BCas and its association with patient survival. Furthermore, we studied the impact of 13q14.2 loss on cancer cells and their surrounding microenvironment. Finally, we examined the extend, to which 13q14.2 loss affects the sensitivity of BCa cells to venetoclax and other BCL2 inhibitors.

## Methods

### Bioinformatics analyses

GISTIC-threshold-by-gene-based copy number (CN) data of The Cancer Genome Atlas (TCGA) pan-cancer cohort (ICGC/TCGA, Nature 2020) and Molecular Taxonomy of BCa International Consortium (METABRIC) BCa cohort (Nature 2012 and Nature Communication 2016) were downloaded from cBioPortal [21]. Per sample, CN status of 13q14.2 was called, including deep loss, loss, neutral, gain, amplification or ambiguous. The latter included samples with breakpoints and hence alternating CN levels within the 13q14.2 cytoband. Of all 1,217 experimentally validated human tumor suppressor genes (TSGs) across the human genome (1,018 protein-coding and 199 non-coding genes) [22], 5 are located in cytoband 13q14.2 (3 protein-coding genes (*RB1*, *ARL11*, *KCNRG*) and 2 non-coding genes (*MIR15A* and *MIR16-1*). In the TCGA-BRCA dataset, the latter three genes are not mutated. *ARL11* is mutated in 2 samples. However, these mutations are not reported as pathogenic or likely pathogenic by ClinVar [23]. In total, 25 out of 1066 TCGA-BRCA samples (2.3%) carry *RB1* mutations, 8 of which (7 nonsense mutations and 1 splice site mutation) are reported as pathogenic or likely pathogenic by ClinVar. Thus, these 8 samples (0.8%) were excluded from analyses. Similarly, in the METABRIC dataset, no mutations were identified in 4 of the 5 genes, whereas 56 out of 2,433 samples (2.3%) carried *RB1* mutations, 9 of which (4 frame-shift mutations causing deletions, 4 splice site mutations and 1 nonsense mutation) are reported as pathogenic or likely pathogenic by ClinVar. Thus, these 9 samples (0.4%) were excluded from analyses. Analyses of CNAs, gene expression, protein expression and clinical data using the TCGA and METABRIC datasets were performed using *R* programming language (version 4.3.3) and the figures were produced using the *ggplot2 R* package, version 3.5.1.

### Survival analysis

Overall survival analyses were performed by comparing BCa patients with 13q14.2 CN loss to those without 13q14.2 loss. The *survival R* package [24] was employed. In addition to all patients together, patient survival was also assessed per molecular subtype. Overall survival data were extracted from the TCGA (Nature 20212) and METABRIC clinical data and matched to CN data by patient ID.

### Mutual exclusivity and co-occurrence analyses

For co-occurrence analysis of 13q14.2 loss and top mutated genes in BCa, we first prepared the data by adding a binary column for 13q14.2 loss, indicating the presence or absence of the event. Mutations in frequently mutated cancer genes were also included, with genes as columns and samples as rows, and mutations represented as binary (1 for presence, 0 for absence). We then conducted Fisher’s exact tests for each gene by looping through each gene, constructing a 2 × 2 contingency table for that gene with 13q14.2 loss. Fisher’s Exact Test returns two values: *p*-value and odds ratio for each test. The odds ratio indicates the strength and direction of the association; values greater than 1 suggest co-occurrence, while values less than 1 suggest mutual exclusivity. For each alteration, the Direction was calculated using the log2 of the odds ratio, multiplied by − log10 (*p* value). Genes with significant p values (<0.05) were kept. Genes with positive − log10 (*p* value) * Direction co-occur with 13q.14 loss, while genes with negative − log10 (*p* value) * Direction are mutually exclusive. The same approach was used to explore co-occurrence and mutual exclusivity of copy number loss of 13q14.2 and copy number loss or gain of other cytobands.

### Differential expression analyses

Differential gene expression analyses were performed using the TGCA BCa dataset. Gene expression levels were compared between samples with 13q14.2 loss (n = 464) and samples without 13q14.2 loss (n = 486). For each gene, gene expression levels were scaled to obtain z-scores per sample. The differences between the median gene expression levels were determined for each gene and adjusted *p* values were calculated using Wilcoxon signed-rank tests followed by Bonferroni-adjustment. Protein expression levels were similarly compared, except that *p* values were adjusted using the Benjamini–Hochberg method [25].

### Pathway enrichment analyses

To identify activated pathways as a consequence of 13q14.2 loss, the top 50 differentially expressed genes, including both up- and downregulated genes, were used and subjected to analyses with MSigDB [26], Reactome [27] and Panther [28]. However, to avoid potential false discovery of pathways, downregulated genes located on chromosome arm 13q were excluded (Fig. 4c, Table S1).

### Deconvolution

Deconvolution of bulk expression of RNA-seq data refers to the computational process of estimating the proportions of different cell types that generate the final aggregate measurement. Similar to multiple linear regression model applied to gene expression (microarray or bulk RNA-seq) data, it can be formulated as [29]:

$${\mathbf{P}}_{j} = {\mathbf{SM}}\cdot{{\varvec{\upbeta}}}j + {\varvec{\epsilon}}$$

where **P**_*j*_ is the measured expression/RNA-seq values from tumor sample *j*, **SM** is a Signature Matrix (SM)—that is, a cell-specific expression profile—and ***β****j* is a vector of mixing fractions for sample *j*, and ${\varvec{\epsilon}}$ represents the noise. Thus, for *P*_*ij*_ being the expression of gene *i* in sample *j*, we have: *P*_*ij*_ = *SM*_*ik*_*β*_*kj*_ in which *SM*_*ik*_ is the averaged expression of gene *i* in cell type *k* and *β*_*kj*_ is fraction of cell type *k* in sample *j*. Deconvolution has gained increasing attention and a wide range of methods have been developed [30]. Here, we utilized a common method known as CIBERSORTx [31]. Bulk RNA-seq data of TCGA and gene expression profiles (GEPs) data of METABRIC were downloaded from cBioPortal. TCGA data were log2-normalized and scaled and METABRIC data were scaled. We estimated cell fractions using either “Input Cell Fractions” module of CIBER-SORTx webtool with default settings or the Docker version of CIBERSORTx with a similar setting. Following the pipeline, we developed for more reliable and reproducible deconvolution [32], we estimate cell fractions using previously created 10 Signature Matrices (SMs) (created using high resolution single-cell RNA sequencing (scRNA-seq) from [33], and then averaged over all 10 estimates. We also perform an analysis on association of loss of 13q14.2 and fraction of cell types enumerated from spatial omics data. We used imaging mass cytometry data for a subset of METABRIC cohort [34], which were downloaded from the https://zenodo.org/record/5850952, and compared fractions of different cell types.

### Cell fractions from spatial omics data

To explore the association between loss of 13q14.2 and TME composition in spatial proteomics data, imaging mass cytometry data of a subset of METABRIC cohort were used [34]. These were downloaded from https://zenodo.org/record/5850952. In this dataset, various markers (38 markers including ER, HER2, HLA-ABC, CD45, CD68, etc.) were employed to annotate a wide range of cell types (32 cell types including different epithelial cells, CD4 and CD8 T cells, macrophages, fibroblasts etc.) in TME (see [34] for details on markers and cell types).

### Drug response analyses

Drug response analyses of 52 BCa cell lines were conducted using the Genomic Drug Sensitivity in Cancer (GDSC) datasets GDSC1 and GDSC2 [35], version 8.4 (June 2022). Genomic features included in the analyses were recurrent mutations and focal CNAs, as previously described [36, 37]. A machine learning approach (elastic net regularization [38]) was applied to identify pharmacogenomic interactions, as determined by effect sizes (Glass’ Δ of IC_50_ values) and associated *p* values. These analyses were performed both genome-wide and pertaining to pharmacogenomic interactions involving 13q14.2 loss only. For direct comparisons between cell lines with CN neutral and loss of 13q14.2, *p* values were determined using non-parametric Mann-Whitney *U* tests. Effect sizes and 95% confidence intervals were determined using point biserial correlation coefficient *r*, with *r* value boundaries between negligible, small, medium and large at 0.10, 0.24 and 0.37, respectively [39].

### Patient-derived xenografts

Drug responses to navitoclax in BCa patient-derived xenografts (PDXs) were evaluated using the area under the curve (AUC), as determined in published work [40]. CN profiling determined by shallow whole-genome sequencing was also described in this study, enabling separation of PDXs into groups with and without 13q14.2 loss.

### Cell culture

Human BCa cell lines MFM223, CAL120 and EVSA-T were purchased from Leibniz-Institut DSMZ-Deutsche Sammlung von Mikroorganismen und Zellkulturen GmbH, who provided the authentication certificate. Human BCa cell lines DU4475, BT474, MCF7, BT20, HCC1187, HCC2218 and HCC1500 were purchased from American Type Culture Collection (ATCC). None of the cell lines used in this study were found in the database of commonly misidentified cell lines maintained by the International Cell Line Authentication Committee and NCBI Bio sample. All cell lines were routinely monitored for mycoplasma contamination (MycoAlertTM mycoplasma detection kit, Lonza Cat#LT07-318). MFM223 and EVSA-T cell lines were maintained in EMEM (ThermoFisher Cat#31095029) media. BT20 was maintained in DMEM (ThermoFisher cat#11965084). DU4475, BT474, MCF7, HCC1187 cell lines were maintained in RPMI-1640 media (ThermoFisher Cat# A1049101). Regardless of the media, all cell lines were supplemented with 10% FBS (ThermoFisher Cat#10500064) and 1% penicillin–streptomycin (Gibco; 10,000 U/mL). The PMC42-ET cell line was generated with BCa patient consent and obtained from a pleural effusion by Dr. Robert Whitehead (Ludwig Institute for Cancer Research, Melbourne, Australia), under appropriate institutional ethics clearance (Institutional Review Board of the Peter MacCallum Hospital, Melbourne) [41, 42]. The PMC42-LA subline was a spontaneous derivative sub-line from the parental PMC42-ET cells by Dr. Leigh Ackland (Deakin University, Melbourne, Australia) [43] and had been well characterized as a spontaneous and unique mesenchymal-epithelial transition (MET) model system, which was observed to have more epithelial features than the parental mesenchymal PMC42-ET [44, 45]. The whole multi-omics profiling was also well characterized recently including G-banding karyotype, whole exome sequencing and incorporating transcriptomes and proteomics data from this cell model system [44]. PMC42 cell lines were maintained in Dulbecco’s modified Eagle’s medium (DMEM) containing glucose (4.5 g/L), L-Glutamine (0.5 g/L) and sodium pyruvate (0.1 g/L) (GibcoTM, Thermo, Catalog number—11885084), and supplemented with 10% fetal bovine serum (FBS; GibcoTM, Thermo, Victoria, Australia) and antibiotics, penicillin and streptomycin (GibcoTM, Life Technologies Catalog number—15140122).

### Drug treatment and cell viability assays

Adherent BCa cell lines were seeded into 96-well plates based on optimal growth rates determined empirically for each line (15% confluency in day 1). 24 hours after plating, cells were dosed with 9 series (3- fold series) of drug concentrations (ABT199, Abcam #ab217298) for 72 h. In case of cell lines in suspension, cells were seeded and dosed with the drugs in the same day. CellTiter-Glo® Luminescent Cell Viability Assay (Promega, #G7571) was added (50% v/v) to the cells, and the plates incubated for 10 min prior to luminescent detection in a Victor 2 microplate reader (Perkin Elmer, Waltham, MA, USA) and the signal was quantified at 595 nm. In case of PMC42 cell lines, cells were treated with drugs (ABT199, ABT737 and ABT263 purchased from Sapphire Bioscience, Australia) and the viability was assessed by the resazurin-based Alamar Blue assay (#R7017, Sigma-Aldrich, St. Louis, MO, USA) and the fluorescence intensity in each well was measured after 90 mins using a bottom-reading fluorescent plate reader (CLARIOstar Plus, BMG LABTECH) with excitation at 544 nm and emission at 590 nm.

## Genotyping

### Cell line identification

The identities of all BCa cell lines employed in this study were confirmed by short tandem repeat (STR) profiling.

### SNP6 microarray analysis

Genomic DNA from BCa cell lines was extracted using the Qiagen Blood & Cell Culture DNA Mini Kit (cat #13323) and subjected to SNP6 microarray analysis by the Australian Translational Genomics Centre (ATGC) at the Translational Research Institute (TRI), Princess Alexandra Hospital, using Illumina InfiniumTM Global Screening Array (GSA). The microarray data were analyzed using open-access GenomeStudio Software in conjunction with the CNVPartition plug-in (version 3.2.0).

### Shallow whole genome sequencing

Shallow whole genome analysis was performed on the genomic DNA from a BCa cell line at the Genomic Core Facility at the Amsterdam UMC, Netherlands. The data were analyzed and visualized using Ab-solute Copy Number Estimation in Bioconductor [42].

### Statistical analysis and reproducibility

Unless otherwise stated, data analyzed by parametric tests are represented by the mean ± SEM of pooled experiments and median ± interquartile range for experiments analyzed by non-parametric tests. The n-values represent the number of independent experiments performed. For each independent *in vitro* experiment, at least three technical replicates were used, and a minimum number of three experiments were performed, to ensure adequate statistical power. Student’s t-test was used to compare data with normal distribution, and non-parametric Mann–Whitney *U* test was used for samples not following a normal distribution. The confidence level used for all the statistical analyzes was of 95% (*α *= 0.05). Two-tailed statistical analysis was applied for experimental design without predicted results, and one-tail for validation or hypothesis-driven experiments. For IC_50_ measurement, data were normalized to percent of control and the relative IC_50_ values were determined using GraphPad Prism Version 9.4.0 software. Statistical analysis was performed by GraphPad Prism software Version 9.4.0 and *R* programming language version 4.3.3.

## Results

### 13q14.2 loss is a recurrent event associated with poor breast *cancer* survival

**Fig. 1:**
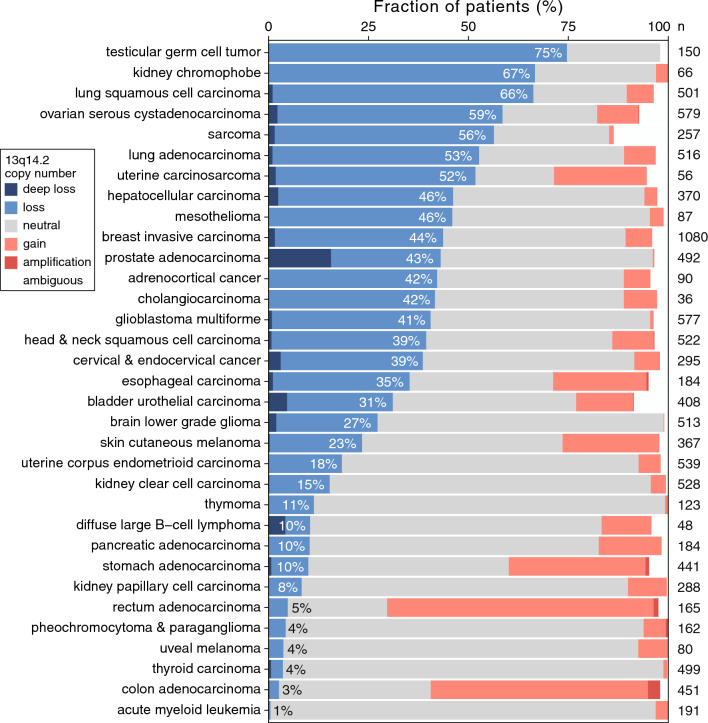
Loss of 13q14.2 is common in breast cancer and other cancer types. Chromosome 13q14.2 copy number status in the TCGA pan-cancer cohort are shown across cancer types. The percentages of samples with copy number loss are shown in each bar. Number of samples in each cancer type (n) are shown

Loss of chromosome cytoband 13q14.2 has emerged as one of the most frequent aberrations in patients with CLL [13]. However, its frequency and clinical implications in other cancers remain largely unexplored. We initially investigated the prevalence of 13q14.2 loss across cancer types in the pan-cancer dataset from TCGA [46]. We found that loss of this cytoband is widespread across cancers, with the highest frequencies respectively observed in testicular, kidney and squamous cell lung cancers, while 44% of BCas harboured this loss (Fig. 1).

**Fig. 2:**
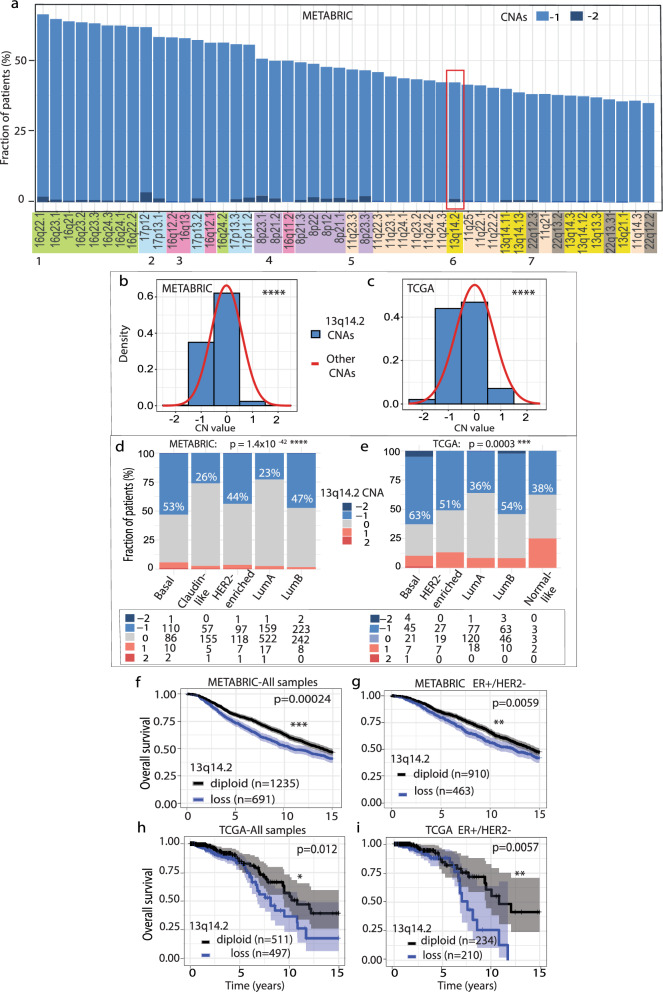
13q14.2 loss is associated with poor overall breast cancer survival. **a** Cytoband-level top 50 most frequent chromosomal losses were assessed using gene-based copy number data from the METABRIC patient cohort. Cytobands on each chromosome are color-coded for easy identification. 13q14.2 is highlighted with a red box. **b**, **c** Histograms comparing the frequencies of copy number alterations of 13q14.2 relative to other regions in the METABRIC (**b**) and TCGA (**c**) cohorts. Statistics: student t-test, *p* < 0.0001: ****. **d**, **e** Relative frequencies of 13q14.2 copy number alterations assessed in the METABRIC (**d**) and TCGA (**e**) cohorts using PAM50 breast cancer classification. The numbers of patients in each subtype are shown in the box below. **f**–**i** Kaplan–Meier plots illustrating the overall survival of breast cancer patients with 13q14.2 loss compared to those with diploid 13q14.2 for all patients within the METABRIC (**f**) and TCGA (**g**) clinical cohorts, and for patients with ER + /HER2- breast cancers in these cohorts (**h**, **i**). − 2: two copy loss, − 1: one copy loss, 0: diploid copy number, + 1: one copy gain, + 2: two or more copy gains. Abbreviations: n: number of samples. *p* values: log-rank test, *p* < 0.05: *, *p* < 0.01: **, *p* < 0.001: ***. ER: estrogen receptor. HER2: human epithelial growth factor receptor. CNA: copy number alteration

Given this notable occurrence of 13q14.2 loss in 44% of BCa patients, we further explored its potential role in BCa. To achieve this, we conducted a series of analyses. First, we examined the prevalence of 13q14.2 loss in comparison to other focal copy number (CN) losses using gene-based SNP6 array data in two large and well-annotated BCa patient cohorts: METABRIC and TCGA. Of note, 13q14.2 loss is part of the 6th most common sub-chromosome arm-level CN loss (Fig. 2a). In TCGA, 13q14.2 is the 7th most common sub-chromosome arm-level CN (Fig. S1a). Loss of 13q14.2 was predominantly heterozygous, with 13q14.2 homozygous loss only observed in a small fraction of patients in both cohorts (<2%).

Secondly, to statistically confirm the previous data, we evaluated the frequency of 13q14.2 CNAs in comparison to other focal CNAs, encompassing both losses and gains, across the METABRIC and TCGA cohorts. Remarkably, in both datasets, the incidence of 13q14.2 loss significantly surpassed that of other losses, whereas its gain was less prevalent compared to gains observed in other regions (*p*<0.0001, *t* test; Fig. 2b, c).

Thirdly, we explored the frequency of 13q14.2 CNAs within distinct histological and molecular BCa subtypes in METABRIC and TCGA, as delineated by the Prediction Analysis of Micro-array 50 classification (PAM50) [47]. Loss of 13q14.2 was consistently more common in ductal than in lobular tumors. However, this difference was only statistically significant in the TCGA cohort (Fig. S1b). Notably, 44-63% of patients classified as basal, HER2-enriched, and luminal B subtypes exhibited 13q14.2 loss, while patients identified as luminal A and Claudin-like subtypes manifested this loss considerably less frequently in both cohorts (p≤0.0003, Chi square test; Fig. 2d, e). Less than 2% of all patients exhibited amplification of 13q14.2 in these cohorts.

Lastly, we utilized the clinical data from the METABRIC and TCGA datasets to explore the impact of 13q14.2 loss on patient overall survival. Patients with 13q14.2 loss demonstrated markedly poorer survival outcomes compared to their diploid counterparts within both METABRIC (Fig. 2f) and TCGA cohorts (Fig. 2g). Further stratification by molecular subtypes showed that this also applied to the clinically relevant ER+/HER- subgroups in both cohorts (Fig. 2h, i). Statistical significance was not reached in the ER-/HER2-, HER2+ or triple-negative BCa (TNBC) subtypes of the METABRIC or TCGA cohorts (Fig. S1c–h).

These collective findings underscore the prevalent occurrence of 13q14.2 loss in BCas, its association with adverse survival outcomes in specific molecular subtypes, and its potential as a clinically relevant biomarker.

### 13q14.2 loss in breast *cancer* is part of a larger repertoire of genomic alterations rather than an isolated event

**Fig. 3:**
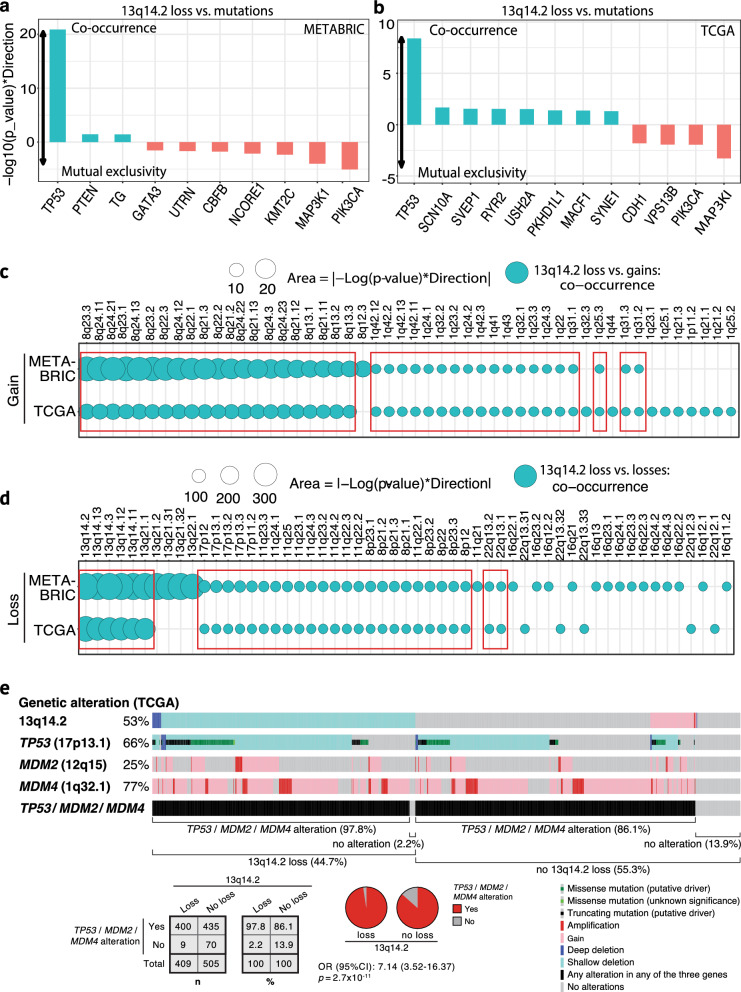
13q14.2 loss accrues concurrently with other genomic alterations in breast cancers. Mutual exclusivity and co-occurrence of 13q14.2 loss with top mutated genes in (**a**) METABRIC and (**b**) TCGA breast cancer cohorts were analyzed. Mutual exclusivity and co-occurrence of 13q14.2 loss with top focal copy number alterations was analyzed in (**c**) METABRIC and (**d**) TCGA breast cancer cohorts. Observations consistent between the cohorts are highlighted with red boxes. Statistics: Fisher’s Exact Test. **e** OncoPrint summarizing the cooccurrences of genomic alterations affecting 13q14.2, *TP53*, *MDM2* and *MDM4* in the TCGA breast cancer cohort. Statistics: Fisher’s exact test with odds ratio (OR) and 95% confidence interval (CI)

In cancer cells, multiple evolutionary pathways are pursued to promote survival and growth. Oncogenic drivers compete under selective pressure, leading to the dominance of certain drivers while others are outcompeted, resulting in mutual exclusivity [4, 48]. However, certain oncogenic drivers may also be selected concomitantly, most likely due to a co-regulatory function during tumorigenesis [48]. To ascertain whether 13q14.2 loss represents an independent event or if it coincides with other genomic alterations in BCas, we performed co-occurrence and mutual exclusivity analyses with both top mutated genes and other recurrent CNAs in TCGA and METABRIC BCa cohorts. Firstly, we conducted an analysis of mutual exclusivity or co-occurrence using Fisher’s exact tests, which involved computing *p*-values and odds ratios for each gene among those with a mutation frequency greater than 2% against the loss of 13q14.2 in both the TCGA and METABRIC cohorts. Mutated genes exhibiting a positive − log10(*p* value) * Direction, such as *TP53*, strongly co-occurred with 13q14.2 loss (Fig. 3a, b). The observed co-occurrence of *TP53* mutations with loss of 13q14.2 is particularly noteworthy, given that *TP53* serves as the gatekeeper of the cell cycle [49]. Loss of the 13q14.2 cytoband, which includes the *RB1* gene that controls the cell cycle, combined with *TP53* mutation, synergistically leads to a significant reduction in the cell’s ability to regulate its growth and repair DNA damage. This identifies a potential treatment target for tumors harboring loss of the 13q14.2 cytoband.

We further performed these analyses in PAM50-based and molecular subtypes of BCa in both cohorts. Notably, 13q14.2 loss co-occurred with *TP53* mutations in the ER+/HER2- molecular subtype in both the TCGA and METABRIC cohorts (Fig. S2a). However, other co-occurrences were inconsistently associated in molecular subtypes in the two cohorts (Fig. S2a, S2b) (Table 1).

**Table 1:** Validated co-occurring and mutually exclusive events with 13q14.2 loss in breast cancer

Breast cancer subtype	Co-occurring (loci on indicated chromosome arms)*	Mutually exclusive (loci on indicated chromosome arms)*	Figures
All breast cancers	*TP53* mutations, 8q gain, 1q gain, 13q loss, 17p loss, 11q loss, 8p loss, 22q loss	*MAP3KI* mutations, *PIK3CA* mutations	Figure 3a-e
ER + /HER2-	***TP53* mutations**, **8q gain**, **13q loss**, 17p loss, 8p loss, **22q loss**	***MAP3K1* mutations**	Fig. S2a, Fig. S3a, c
ER-/HER2-	**10p gain**, **5q loss**, **14q loss**, **4p loss**, **15q loss**, 17p loss		Fig. S3a, c
HER2 +	17p loss, **11q loss**, 8p loss, **1p loss**, **13q loss**, **16q loss**		Fig. S3c, d
Luminal A	**8q gain**, 17p loss, 8p loss, **22q loss**		Fig. S3b, d
Luminal B	17p loss		Fig. S3d
Basal	**1q gain**, 17p loss, **14q loss**, **15q loss**, **4p loss**, **5q loss**		Fig. S3b

*13q14.2 loss co-occurring and mutually exclusive events specific for 2 (sometimes overlapping) subtypes are highlighted in **bold**; those specific for a single subtype are shown in **bold underlined**

Conversely, mutations in *MAP3K1* and *PIK3CA* showed the strongest mutual exclusivity with 13q14.2 loss in both cohorts (Fig. 3a, b). Further analyses in subtypes revealed that the mutual exclusivity between *MAP3K1 and 13q14.2* loss was present in the ER+/HER2- subtype across both cohorts (Fig. S2a) (Table 1). However, mutual exclusivity was not consistently observed for *MAP3K1* or *PIK3CA* in other subtypes across the METABRIC and TCGA cohorts (Fig. S2a, S2b). Thus, 13q14.2 loss consistently co-occurs with *TP53* mutations in BCa and specifically in ER+/HER2- BCas and 13q14.2 loss is consistently mutually exclusive with mutations in *PIK3CA* and *MAP3K1* in BCa, while in subtypes, mutual exclusivity is only consistently observed for *PIK3CA* mutations in the ER+/HER2- subtype.

Secondly, we conducted a similar analysis to assess the co-occurrence or mutual exclusivity of 13q14.2 loss with the gain or loss of other top altered cytobands in both the TCGA and METABRIC cohorts. Notably, loss of 13q14.2 often coincides with gains of 8q12-q24 and 1q22-q43 (Fig. 3c), as well as with losses of cytobands adjacent to 13q14.2 and 17p11-p13, 11q22-q25, 8p12-p23 and 22q13.1-q13.2 (Fig. 3d) across both patient cohorts. We further conducted these analyses across molecular and PAM50-based BCa subtypes. Notably, 13q14.2 loss consistently strongly co-occurred with 8q22-q24 gains in the ER+/HER2- and Luminal A subtypes in both the TCGA and METABRIC cohorts, while gain of 10p15.1 and 1q24.2 co-occurred with 13q14.2 loss in ER-/HER2- and basal BCas, respectively (Fig. S3a, S3b) (Table 1). Remarkably, 9 chromosome cytoband losses consistently co-occurred with 13q14.2 loss in BCa subtypes with some subtype-specificity, including loss of 17p11-p13, 11q22-q25, 8p21-p23 and 22q12-q13 in ER+/HER2- subtype, loss of loci on 5q, 14q, 4p and 15q in ER-/HER2- BCas, and loss of loci on 17p, 11q and 8p in HER2+ BCas (Fig. S3c, S3d) (Table 1). In Pam50 classification, losses of 17p12-p24, 11q21-q24 and 22q11-q13 coincided with 13q14.2 loss in luminal A tumors, while loss of 16q24 occurred in HER2+ BCas and loss of loci on chromosomes 14q, 15q, 4p and 5q was exclusively observed in basal cancers (Fig. S3d) (Table 1).

It is noteworthy that the tumor suppressor gene *TP53* (17p13.1) and its negative regulator *MDM4* (1q32.1) are located within the co-occurring loci with 13q14.2 loss. To further explore this, we conducted additional analyses of the genomic alterations involving these genes in TCGA BCa samples, comparing those with and without 13q14.2 loss. This revealed that somatic alterations in the *TP53*, *MDM2* and/or *MDM4* genes, involving both mutations and CNAs, occurred 11.7% more frequently in cases where 13q14.2 was lost (Fig. 3e; *p *= 2.7 × 10^-11^, Fisher’s exact test). This suggests a potential functional relationship between the loss of 13q14.2 and p53 pathway inactivation.

Collectively, these observations suggest that 13q14.2 loss may not act alone but interacts with other genomic alterations in BCa (Table 1).

### 13q14.2 loss affects breast *cancer* cells and their tumor microenvironment

**Fig. 4:**
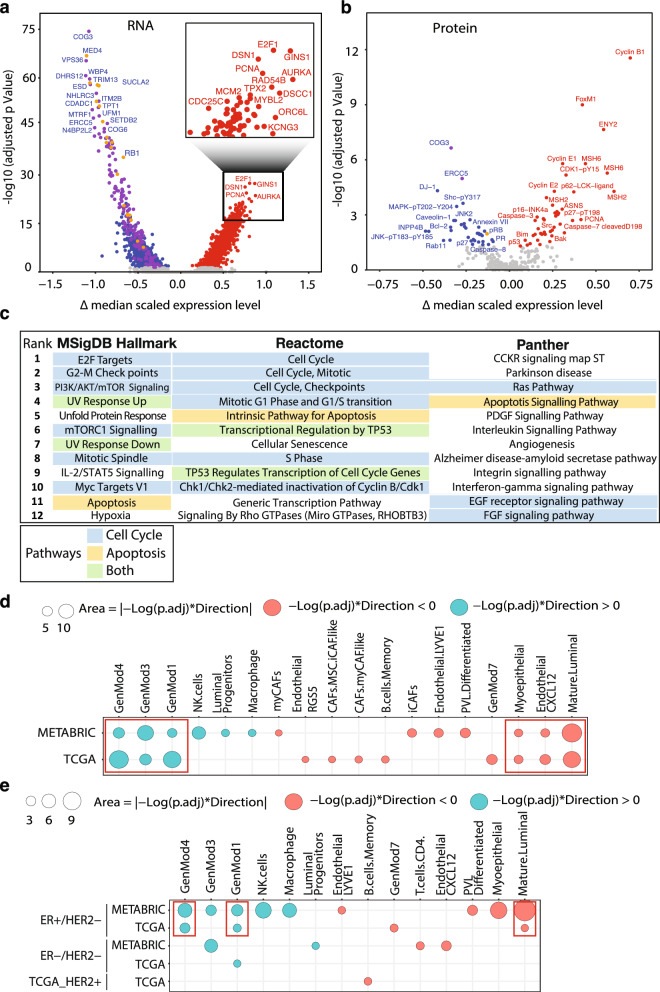
13q14.2 loss activates cell cycle and apoptosis-related pathways in cancer cells and affects the tumor microenvironment. **a** Volcano plot demonstrating differential gene expression analysis in TCGA patient samples with and without 13q14.2 loss. Blue dots: genes significantly downregulated. Red dots: genes significantly upregulated (i.e., adjusted *p* value < 0.05). Orange circles: genes on the 13q14.2. Purple circles: all other genes on chromosome arm 13q. **b** Volcano plot demonstrating differential (phospho-)protein expression analysis in TCGA patient samples with and without 13q14.2 loss. Blue dots: proteins significantly downregulated. Red dots: proteins significantly upregulated (i.e., adjusted *p* value < 0.05). Orange circles: proteins whose genes reside on 13q14.2. Purple circles: all other proteins whose genes reside on chromosome arm 13q. **c** Pathway analyses performed on the upregulated genes and proteins in TCGA samples with 13q14.2 loss using three different pathway enrichment tools, including MSigDB hallmarks, Reactome and Panther. **d** Comparison between fractions of cell types in tumors with 13q14.2 loss versus 13q14.2 diploid in the METABRIC and TCGA cohorts. Observations consistent between the cohorts are highlighted with red boxes. **e** Comparison between fractions of cell types in tumors with 13q14.2 loss versus 13q14.2 diploid in clinical molecular subtypes in the METABRIC and TCGA cohorts

To study the impact of 13q14.2 loss on cancer cells, we conducted differential gene expression analysis (DEA) using mRNA data from patient samples with 13q14.2 loss compared to those diploid for this region, utilizing the TCGA BCa dataset. As expected, the expression of multiple genes located in the 13q14.2 region were significantly downregulated (Fig. 4a, orange circles). Interestingly, other genes located on the 13q chromosome arm were also downregulated, suggesting that a considerable number of patients have lost larger fractions of the 13q chromosome arm in addition to 13q14.2 loss (Fig. 4a, purple circles). In contrast, multiple genes involved in cell cycle-related pathways were remarkably upregulated, including but not limited to *E2F1*, *DSN1*, *GINS1*, *PCNA*, *AURKA*, *TPX2* and *DSCC1* (Fig. 4a, red circles).

To complement this gene expression analysis, we also performed differential protein expression analyses in the same patient samples, where available. It is worth noting that the number of statistically significant downregulated (phospho-) proteins on 13q14.2 (n = 1) and 13q (n = 3 including the one on 13q14.2) was considerably lower than at the mRNA level. This difference can be attributed to the fact that fewer (phospho-)proteins were analyzed, and post-transcriptional compensatory mechanisms that may affect protein levels. Consistent with the gene expression data, protein levels of many cell cycle-related proteins were significantly increased, including CyclinB1, FoxM1, and CyclinE1. Additionally, we observed upregulation of several pro-apoptotic components, including Caspase-7-cleaved D198, BAK, BIM, and Caspase-3 (Fig. 4b, red circles). Conversely, anti-apoptotic proteins like BCL2 were downregulated (Fig. 4b).

These observations prompted us to perform pathway analysis using the combined mRNA and protein differential expression data. To minimize potential biases from downregulated genes on 13q14.2 caused by CN loss, which may not reflect true functional differences, we focused our analysis on upregulated genes and proteins, as well as downregulated genes, excluding those located on 13q. By analyzing the top significantly upregulated and downregulated mRNAs and proteins, using sources including ’MSigDB Hallmark’, ’Reactome’ and ’Panther’, we identified two prominent upregulated pathways: ’Cell cycle’ and ’Apoptosis’ (Fig. 4c, Table S1). Interestingly, the loss of the 13q14.2 region is associated with upregulation of the PI3K/AKT/mTOR and p53 pathways.

To explore the potential impact of 13q14.2 loss on the cellular composition in the TME, we estimated the fractions of different cell types within the breast TME. Employing our established pipeline [32], we utilized high-resolution scRNA-seq data [33] as a reference for the breast TME and employed CIBERSORTx as a deconvolution method [31]. The scRNA-seq data encompassed a diverse array of cell types, including cancer cells divided into seven recurrent gene modules (GenMod1-7). Each module is enriched in specific biological pathways, such as TNFα, apoptosis and p53 signaling for GenMod1; IFN-α/γ signaling, antigen presentation, apoptosis and EMT for GenMod3 and cell cycle and proliferation for GenMod4 [33]. Our analysis revealed that, in both the TCGA and METABRIC cohorts, loss of 13q14.2 resulted in a higher fraction of GenMod1, GenMod3 and GenMod4, among others characterized by proliferation (GenMod4), apoptosis (GenMod1, GenMod3) and key signaling pathways (TNFα, IFN-α/γ) affecting immune cells in the TME (GenMod1, GenMod3) (Fig. 4d). This finding strongly aligns with our DEA, which indicated that loss of 13q14.2 identified enrichment of cell cycle-related and pro-apoptotic proteins and pathways (Fig. 4a–c). Additionally, we observed a significant decrease in the fractions of healthy epithelial and endothelial cells, such as mature luminal cells and myoepithelial cells, associated with 13q.14.2 loss, consistent with the presence of fewer differentiated cells (Fig. 4d). Furthermore, 13q14.2 loss is associated with a higher fraction of natural killer (NK) cells and macrophages in METABRIC cohort. While not validated in the TCGA cohort, this is consistent with increased TNFα, IFN-α/γ signaling associated with enriched GenMod1 and GenMod3 modules validated in both cohorts (Fig. 4d). Analysis of the cell fractions enumerated from spatial omics data in the METABRIC cohort confirmed a positive association between 13q14.2 loss and an increased number of macrophages (Fig. S4a). Additionally, an increase in the population of macrophages and NK cells was specifically observed in the ER+/HER2- subtype within the METABRIC cohort (Fig. 4e). In the PAM50 classification, the NK cell population was increased exclusively in the luminal B subtype of the METABRIC cohort (Fig. S4b).

These findings collectively suggest that 13q14.2 loss contributes to alterations in the composition of the TME, favoring the proliferation of cancer cells.

### Loss of 13q14.2 sensitizes breast *cancer* cells to BCL2 inhibitors

**Fig. 5:**
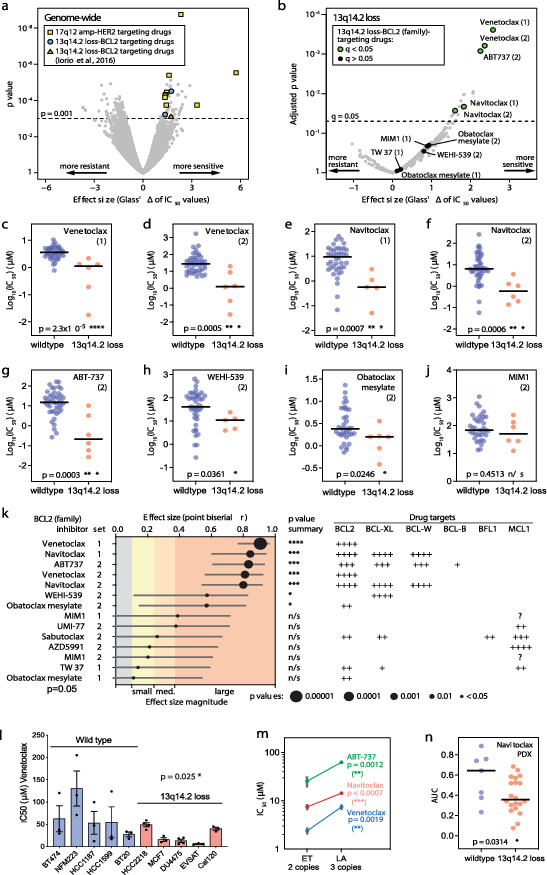
13q14.2 loss is associated with sensitivity to BCL2 inhibitors in breast cancer. **a** Volcano plot showing an elastic model to evaluate the association of 410 established genomic features (high confidence cancer gene mutations and recurrent focal CNAs) with IC_50_ values of 438 drugs across 52 breast cancer cell lines. Selected established (involving HER2) and new pharmacogenomic interactions (involving 13q14.2 loss) are highlighted. Cut-offs: *p* value: 0.001, FDR: 0.25, Glass’ ∆ effect size: 1.4. **b** Volcano plot evaluating the associations between 13q14.2 loss and all drugs in a panel of 673 tested drugs from two independent screens (1 and 2, respectively), including some drugs tested in both screens. The number of unique drugs is smaller. Cut-offs: adjusted *p* value (*q* value): 0.05, FDR: 0.25, Glass’ ∆ effect size: 1.4. Pharmacogenomic interactions involving BCL2 inhibitors are highlighted in green (q < 0.05) and black (q < 0.05). **c**–**j** Scatter dot plots comparing the log10 (IC_50_) of BCL2 inhibitors from the two screens in BCas cell lines with wild type 13q14.2 (blue) and 13q14.2 loss (red). *p* values: Two-tailed Mann–Whitney U tests. *p value < 0.05, **p value < 0.01, ***p value < 0.001, ****p value < 0.0001. **k** Left: Effect size magnitudes for each of the BCL2 inhibitors are shown in different colors across all BCa cell lines possessing 13q14.2 loss. The circle sizes correspond the *p* values. This analysis includes duplicates, i.e., same drugs tested in both screens, included in dataset 1 (set = 1) and dataset 2 (set = 2). Right: Target proteins of individual drugs are shown. Magnitudes of inhibitory effects are shown by increasing numbers of ’+’ symbols; ?: unknown effect. **l** IC_50_ sensitivity to venetoclax was assessed in 5 breast cancer cell lines with wild type 13q14.2 and 5 breast cancer cell lines with 13q14.2 loss. Statistics: one-tailed Mann–Whitney U test. **m** IC_50_ sensitivity to venetoclax, navitoclax and ABT-737 was assessed in a breast cancer cell line with two copies of chromosome 13 (PMC42-ET) and a PMC42-ET-derived breast cancer cell lines with an extra copy of chromosome 13 (PMC42-LA). Vertical grey bars show the range of IC_50_ values determined in three independent replicate experiments with the median values shown as dots. *p* values: Paired t tests. **n** Dot plot graph comparing the response to navitoclax between breast cancer patient-derived xenografts with 13q14.2 loss and their wildtype counterparts. Data extracted from (Bruna et al. 2016). AUC: area under the ROC curve. *P* value: Two-tailed Mann–Whitney *U* test

Following a Sanger Institute pipeline [35, 36], we previously applied a machine learning approach to a pan-cancer drug screen dataset to identify mutations and focal or chromosome arm-level CNAs associated with increased drug sensitivity or resistance [37]. Here, we leveraged an expanded version of this dataset to apply this approach on a BCa-specific subset of these data, including 30,394 half-maximum inhibitory concentration (IC_50_) values from 542 unique drugs across 52 BCa cell lines. Notably, using this unbiased approach, we identified a novel CNA-drug association with robust impact: loss of 13q14.2 strongly associates with increased sensitivity of BCa cells to *BCL2*-targeting drugs (Fig. 5a, blue circles). This association was also identified by the Sanger Institute analysis (Fig. 5a, orange triangle) using a prior version of the dataset, albeit at a lower statistical significance level [36]. We note that our analysis also identified genomic amplification of the *HER2* gene, located on chromosome 17q12, as strongly associated with sensitivity to HER2-targeting drugs in BCa cells (Fig. 5a, yellow squares). Thus, identification of this well-established interaction [50] validates our approach.

To further explore the association between 13q14.2 loss and BCL2 inhibitors, we assessed the effect sizes and sensitivities of 673 tested drugs to 13q14.2 loss in BCa cell lines, identifying those with the most significant impact. Intriguingly, BCL2 inhibitors, including venetoclax, ABT737, and navitoclax, displayed the most significant associations with increased sensitivity in BCa cell lines with 13q14.2 loss (Fig. 5b, green circles). However, we noted that 13q14.2 loss did not statistically significantly associate with other BCL2 inhibitors. In addition, 13q14.2 loss did not statistically significantly associate with resistance to any of the 673 drugs (Fig. 5b).

Classifying the BCa cell lines into two groups, those with loss of 13q14.2 and those wild type (diploid) for this region, we then compared their sensitivities to these 7 BCL2 inhibitors. This showed that loss of 13q14.2 resulted in significantly lower IC_50_ values (indicating greater sensitivity) for venetoclax, navitoclax, ABT-737 and WEHI-539, including in two independent screens for the former two (Fig. 5c–i), but not for MIM1, sabutoclax, TW37, AZD5991 and UMI-77 (Fig. 5j, Fig. S5a–f). We observed a marginally increased sensitivity for obatoclax in one screen (Fig. 5i) but not in another screen (Fig. S5c).

To further characterize the sensitivities of BCa cell lines with 13q14.2 loss to BCL2 inhibitors, we determined the effect size magnitude of each inhibitor. Venetoclax exhibited the largest effect size with the lowest *p* value < 0.0001, followed by navitoclax and ABT-737 with *p* values < 0.001. WEHI-539 and obatoclax ranked third with *p* values < 0.01 and < 0.05, respectively (Fig. 5k, left). Notably, comparison of the selective inhibitory effects of each drug towards different BCL2 family member proteins strongly suggests that these increased sensitivities are primarily, if not exclusively, due to inhibition of BCL2 protein, rather than other BCL2 family member proteins (Fig. 5k-right).

To validate the increased sensitivity of BCa cells with 13q14.2 loss to BCL2 inhibitors *in vitro*, we treated a panel of 10 BCa cell lines with and without 13q14.2 loss venetoclax, the most specific inhibitor of *BCL2*. The presence or absence of 13q14.2 loss and its approximate size within the genome was initially confirmed by SNP6 microarray (Fig. S6b–h) and shallow sequencing (Fig. S7). Subsequently, the cell lines were treated with serial log*10*-fold concentration dilutions of venetoclax, and their viability was measured after 72 hours. Importantly, cells with 13q14.2 loss exhibited significant sensitivity to venetoclax compared to their wildtype counterparts (Fig. 5l, Fig S5g–p) (One-tailed Mann-Whitney u-test, *p* value = 0.025). It is worth noting that the response to venetoclax varied across the cell lines harboring a loss of 13q14.2 with EVSA-2 being the most sensitive and HCC2218 the least sensitive to venetoclax (Fig. 5l). The variation was also seen in the cell lines with diploid or amplified 13q14.2 with MFM223 the most resistant and BT20 the least resistant cell lines to venetoclax (Fig. 5l).

To evaluate whether the presence or absence of BCL2 protein affects the association between loss of the 13q14.2 region and sensitivity to venetoclax in cell lines, we measured the protein levels of *BCL2*. However, our data suggest that the relationship between 13q14.2 deletion and venetoclax sensitivity appears to be independent of *BCL2* expression (Fig. S5q).

To investigate whether amplification of this region influences the response to BCL2 inhibitors, we employed two BCa cell lines: PMC42-ET, possessing two copies of chromosome 13, and PMC42-LA, derived from PMC42-ET with three copies of chromosome 13 [51] (Fig. S8a, b). We evaluated the chemotherapeutic sensitivities of PMC42-ET and PMC42-LA to Venetoclax (ABT-199) and two pan-specific *Bcl-2/Bcl-XL* inhibitors, ABT737 and Navitoclax (ABT263). The IC_50_ values for each drug were determined by treating the cell lines with serial 10-fold dilutions of the drugs and measuring their viability after 72 hours. Our drug assays consistently showed that PMC42-LA cells, with an extra copy of chromosome 13, exhibited significantly higher resistance to all three drugs compared to PMC42- ET (Fig. 5m, Fig. S8c–e). Consequently, our observation that cells harboring an extra copy of chromosome 13 exhibit increased resistance to three BCL2 inhibitors aligns with our findings that the loss of the 13q14.2 region enhances the sensitivity of BCa cell lines to these drugs.

Considering that patient-derived xenograft models (PDXs) more faithfully replicate the molecular characteristics and tumor environment of patients, we expanded our analysis from cell lines to data obtained from a biobank of BCa patient-derived xenograft explants [40]. We aimed to determine the effect of 13q14.2 loss on the sensitivity of PDXs to navitoclax compared to their wildtype counterparts. Notably, the increased sensitivity to the drug resulting from 13q14.2 loss was also evident in these PDX models (Fig. 5n), providing further support for the significant role of this CNA in the treatment response to BCL2 inhibitors.

These data collectively suggest that loss of 13q14.2 enhances the therapeutic response to BCL2 inhibitors. Consequently, loss of 13q14.2 could potentially serve as a predictive biomarker for the efficacy of BCL2 inhibitors in treating BCa.

## Discussion

While *RB1* on the 13q14.2 locus has been extensively studied due to its established tumor suppressor function [16, 52, 53], the 13q14.2 region encompasses additional genes that may significantly influence the development and progression of BCa beyond *RB1*. This perspective is supported by both publicly available sequencing data and our own research, which demonstrate the variable extent of 13q14.2 losses across various BCa cell lines and patient samples (Fig. S9). The impact of other potential genes in this region is particularly underscored by studies identifying the concurrent loss of *RB1* and *lysophosphatidic Acid Receptor 6 (LPAR6)* at the 13q14.2 locus, a combination that has been shown to increase the proliferation rate of BCa cells [52, 54]. Moreover, the relationship between larger CNAs and increased disease aggressiveness, along with their significant implications for clinical outcomes, has been a subject of intensive research focus [55–57]. In hematological cancers such as CLL, the loss of chromosome 13q14 is a predominant event and serves as a key biomarker, contributing to improved prognosis and extended patient survival [13]. Our findings revealed that in solid tumors, recurrent loss of 13q14.2 is a defining feature of the genomic landscape, underscoring its significance in tumor pathogenesis. We focused on the prominence of chromosome 13q14.2 loss as a prevalent chromosomal aberration across BCa subtypes, occurring in over one-third of all BCas cases from the TCGA and METABRIC cohorts. The frequent loss of a single copy of chromosome 13q14.2, compared to other CNAs, suggests that this loss may confer a selective advantage for tumor cells, particularly in the context of p53 pathway inactivation. This may ultimately contribute to poorer overall survival outcomes for patients.

Paradoxically, our work revealed that loss of 13q14.2 is associated with upregulation of both cell cycle and pro-apoptosis pathways (Fig. 4a–e). Since *RB1* is located within 13q14.2, conceptually, loss of *RB1* alone could be sufficient for causing both. However, in non-tumorigenic mammary epithelial cells, RB1 knockdown has been shown to cause reversible growth arrest, rather than apoptosis [58]. Thus, our observed effects associated with 13q14.1 loss are likely caused by loss of other genes on 13q14.2, such as *DLEU2* or *TRIM13*, as supported by some studies [59–61]. Alternatively, these may be caused by the simultaneous loss of multiple genes on 13q14.2. Further studies are required to elucidate the precise mechanism.

Recent findings highlight a significant mutual exclusivity between common mutations and CNAs in cancers. For example, in pancreatic cancer, *kirsten rat sarcoma viral oncogene homologue (KRAS)* mutations are mutually exclusive with chromosome 18q gains, both targeting overlapping signaling pathways [62]. Similarly, in our BCa study, mutual exclusivity between *PIK3CA* and *MAP3K1* mutations and the loss of 13q14.2 raises intriguing questions about the functional redundancy or compensatory mechanisms within cancer cells. *PIK3CA*, encoding a subunit of PI3K, directly influences the PI3K/AKT pathway, a critical route for promoting oncogenic activity, including cell growth and survival [63]. In our differential expression analyses, we identified activation of the PI3K/AKT pathway in response to 13q14.2 loss. This suggests that the loss of 13q14.2 mimics the activating effects of *PIK3CA* mutations. Such redundancy underscores the cell’s ability to maintain crucial oncogenic signaling by alternative genomic routes, which may compensate for the absence of mutations in key pathway genes like *PIK3CA per se*.

The co-occurrence of both *TP53* CN loss and mutations with 13q14.2 loss in BCa samples indicates a complex regulatory mechanism involving both alleles of *TP53*. In many cancers, *TP53* function is disrupted. This may occur via a range of mechanisms, including *TP53* mutation, *TP53* CN loss, or gain or amplification of genes encoding p53-antagonizing proteins, like *MDM2* and *MDM4* [64, 65]. For instance, gain of chromosome 1q, which harbors the *MDM4* gene and occurs in many BCas, was recently shown to be largely mutually exclusive with *TP53* mutations, but associated with downregulation of p53 target genes [62]. Moreover, a recent study showed that *TP53* loss of heterozygosity (LOH) results in genomic instability, which leads to chromosome 13 loss, along with other CNAs, during the progression of pancreatic ductal adenocarcinoma [66]. These notions are consistent with our observation that 13q14.2 loss statistically significantly co-occurs with *TP53*-inactivating genomic events and support our hypothesis that the loss of 13q14.2 in BCa could be a downstream consequence of initial *TP53* pathway-impairing genomic alterations.

CNAs as a ubiquitous feature of cancers have been attributed to the imbalance in transcriptomic and proteomic landscapes of tumor cells [67, 68]. We observed a significant upregulation of several genes and proteins associated with cell cycle and pro-apoptotic pathways, as well as downregulation of anti-apoptotic genes, such as *BCL2*, in patient samples harboring 13q14.2 loss. We envisage two possible scenarios for these observations: 1. Apoptosis, a critical cell death program, plays a dual role in cancer. While primarily known for its tumor-suppressing effects [69, 70], apoptosis also has pro-oncogenic properties. The pro-oncogenic property is mainly due to its non-cell-autonomous effects, particularly involving the interaction with tumor-associated macrophages in the tumor microenvironment [70–72]. This correlates with our findings that demonstrate the positive impact of 13q14.2 loss on the macrophage populations within the TME. When apoptotic tumor cells are cleared by macrophages—a process known as efferocytosis—it can create an anti-inflammatory and pro-tumorigenic environment [73]. This process often results in the secretion of cytokines and growth factors that can further promote tumor growth and metastasis. 2. As demonstrated in our study, additional genomic or pathway alterations, such as alterations in *TP53* or activation of the *PI3K/AKT* pathway—both known for inhibiting apoptosis and promoting cell survival—often coincide with 13q14.2 loss. Of note, these alterations can counteract the effects of activated apoptotic pathways, enabling cancer cells not just to survive but to thrive, enhancing their malignant potential [74, 75].

Our data suggest that 13q14.2 loss in BCas may confer a selective advantage by promoting oncogenic behaviors, enhancing tumor growth and survival. Yet, intriguingly, this same genomic alteration also introduces vulnerabilities that can be exploited for therapeutic purposes. Our research has revealed that the loss of 13q14.2 sensitizes BCa cells to BCL2 inhibitors, offering a unique, paradoxical opportunity for targeted intervention. This dual impact of 13q14.2 loss highlights the complex, interwoven regulatory networks within cancer cells, where a single genomic alteration can both bolster cancer cell survival and introduce critical susceptibilities. It is worth noting that the BCL2 inhibitor venetoclax is recognized for its dual mechanism of action: it not only inhibits the BCL2 protein but also induces metabolic rewiring independently of BCL2 inhibition [76]. In our studies, we have observed a BCL2-independent response to venetoclax in BCa cell lines, along with a variable response in cell lines harboring 13q14.2 loss. This suggests that partial or extensive losses within this region, which involve a varying number of genes, might play a role in modulating drug sensitivity.

While our study highlights the relevance of 13q14.2 loss in BCa, it also uncovers a significant gap. Notably, there is a lack of an isogenic *in vitro* model that specifically targets 13q14.2 loss. This is crucial for excluding cell-autonomous effects of other genomic alterations present in cell lines and for a detailed mechanistic study of the impact of 13q14.2 loss on BCa cell biology and drug response. The recent adoption of CRISPR-CAS9 technology to facilitate the development of stable cell lines with specific CNAs [77, 78] can enable more precise investigations into the biological consequences of 13q14.2 loss.

## Conclusion

Our research into the loss of chromosome 13q14.2 in BCa reveals its profound impact on both the genomic and therapeutic landscapes of the disease. This chromosomal aberration is associated with poor patient survival, likely by promoting oncogenic traits, such as a proliferative phenotype. However, it also introduces a specific vulnerability to BCL2 inhibitors, which may be harnessed as a strategy in cancer therapy. This dual role highlights the importance of understanding genomic alterations for developing targeted cancer treatments.

## Supplementary Information

Additional file 1

Additional file 2

Additional file 3

## Data Availability

All *R* code required to replicate the analyses are available at https://github.com/Paris-Shahrouzi/Parastoo-Shahrouzi-13q14.2-loss.git. All data generated or analyzed during this study are included in this published article [and its supplementary information files]. The datasets supporting the conclusions of this article are available in the [TCGA PanCancer Atlas study and BRCA METABRIC (2016)], [https://www.cbioportal.org]. For bioinformatics analyses, *R* programming language version 4.4.0 was employed. The graphical abstract was created with BioRender.com.

## Abbreviations

BCa: Breast cancer
CNA: Copy number alterations
CIN: Chromosomal instability
CLL: Chronic lymphocytic leukemia
CRISPR-CAS9: Clustered regularly interspaced short palindromic repeats and CRISPR-associated protein 9
DAE: Differential expression analysis
GEP: Gene expression profile
LOH: Loss of heterozygosity
MET: Mesenchymal-to-epithelial transition
METABRIC: Molecular taxonomy of breast cancer international consortium
PDX: Patient derived xenograft
PAM50: Prediction analysis of microarray 50
SM: Signature matrix
TCGA: The cancer genome atlas
TME: Tumor microenvironment
